# 

**Published:** 1914-05-09

**Authors:** 


### Gifts and Bequests.

The Chelsea Hospital for Women has received £100 from the late Mrs. Jane J. Singleton.

Mr. Samuel Massey, of Manchester, has left the residue of his property for distribution between, among others, the Manchester Royal Infirmary, the Manchester Eye Hospital, and the Manchester Hospital for Diseases of the Throat and Chest.

Mr. Thomas Edwin Crocker, of Princes Gate, S.W., has left £500 each to the Tavistock (Devon) Cottage Hospital, King's College Hospital, the Wimbledon Cottage Hospital, and the Nelson Hospital, Merton. He empowered his trustees to apply a sum not exceeding £5,000 for such charitable purposes as they may see fit within five years following his decease.

Rates of Subscription: Twelve months, 6/6; Six months, 4/-; Three months, 2/-, post free to any address in the United Kingdom. Abroad, Twelve months, 8/8; Six months, 5/-; Three months, 2/6, post free.—Address The Subscription Dept., "The Hospital," The Hospital Building, 28/29 Southampton Street Strand, London, W.C.

